# Neutron total scattering investigation on the dissolution mechanism of trehalose in NaOH/urea aqueous solution

**DOI:** 10.1063/4.0000065

**Published:** 2021-02-10

**Authors:** Hong Qin, Changli Ma, Sabrina Gärtner, Thomas F. Headen, Taisen Zuo, Guisheng Jiao, Zehua Han, Silvia Imberti, Charles C. Han, He Cheng

**Affiliations:** 1 Institute of High Energy Physics, Chinese Academy of Sciences (CAS), Beijing 100049, China; 2 Spallation Neutron Source Science Center, Dongguan 523803, China; 3 University of Chinese Academy of Sciences, Beijing 100049, China; 4 STFC ISIS Facility, Rutherford Appleton Laboratory, Didcot OX11 0QX, United Kingdom; 5 Institute for Advanced Study, Shenzhen University, Shenzhen 508060, China

## Abstract

Trehalose is chosen as a model molecule to investigate the dissolution mechanism of cellulose in NaOH/urea aqueous solution. The combination of neutron total scattering and empirical potential structure refinement yields the most probable all-atom positions in the complex fluid and reveals the cooperative dynamic effects of NaOH, urea, and water molecules in the dissolution process. NaOH directly interacts with glucose rings by breaking the inter- and intra-molecular hydrogen bonding. Na^+^, thus, accumulates around electronegative oxygen atoms in the hydration shell of trehalose. Its local concentration is thereby 2–9 times higher than that in the bulk fluid. Urea molecules are too large to interpenetrate into trehalose and too complex to form hydrogen bonds with trehalose. They can only participate in the formation of the hydration shell around trehalose via Na^+^ bridging. As the main component in the complex fluid, water molecules have a disturbed tetrahedral structure in the presence of NaOH and urea. The structure of the mixed solvent does not change when it is cooled to −12 °C. This indicates that the dissolution may be a dynamic process, i.e., a competition between hydration shell formation and inter-molecule hydrogen bonding determines its dissolution. We, therefore, predict that alkali with smaller ions, such as LiOH, has better solubility for cellulose.

## I. INTRODUCTION

Cellulose is one of the most abundant natural polymers in the world. For example, its content in wood is 33%–51%, its content in flax reaches 63%–71%, and its content in cotton is an amazing 83%–95%. Each year, the cellulose produced by plants around the world can reach 180 × 10^9^ tons.^1^ Compared with petroleum polymers, cellulose has many excellent characteristics such as being renewable, pollution-free, and low cost, so the use of cellulose instead of fossil products has great potential. However, to transform natural cellulose into regenerating cellulose with good performance, it needs to be dissolved first. The intra- and inter-molecular hydrogen bonds make the cellulose tightly bound; it is, thus, very difficult to dissolve in common solvents.^2–4^

Traditionally, N-methylmorpholine-N-oxide (NMMO), ionic liquids, NaOH/CS_2_, and LiBr/H_2_O are used to dissolve cellulose.^4^ In the early 2000s, the group of Professor Lina Zhang in Wuhan University proposed to use NaOH/urea aqueous solution to dissolve cellulose. A maximum of ∼8 wt. % cellulose can be dissolved in this green solvent within 2 min at −12 °C.^5–7^ This yield is too low for industrial production purposes. Increasing the efficiency requires a detailed understanding of the dissolution mechanism.

Nuclear magnetic resonance (NMR), transmission election microscopy (TEM), wide angle x-ray diffraction (WAXD), and small angle neutron scattering technologies (SANS), amongst others, have been used to study the dissolution mechanism.^8,9^ Based mainly on NMR observations in energy space, the current explanation is that NaOH directly interacts with cellulose, and urea forms a hydration shell around this “NaOH hydrogen bonded cellulose,” forming an inclusion complex (IC).^8–13^ Unfortunately, no molecular image has been derived so far, because the atomic structure in the complex fluid has not been recorded and analyzed directly.

In this work, we used trehalose as a model solute for cellulose, and neutron total scattering as the main tool to study the dissolution mechanism. Trehalose has similar glucose rings to cellulose and is one of the disaccharide molecules with pyranose rings that have no reducibility. Neutron total scattering, combined with empirical potential structure refinement (EPSR), is a nondestructive method to determine the most-probable all-atom positions in the complex fluid (we call the four-component system, i.e., trehalose in urea/NaOH aqueous solution, complex fluid thereafter).

The role of each component, i.e., NaOH, urea, and water, as well as temperature, in the dissolving process can be determined. It is found that NaOH, urea, and water work cooperatively to dissolve trehalose. Both direct and indirect interactions coexist. NaOH directly interacts with trehalose, while urea cannot. Note that cellulose dissolves at −12 °C and precipitates at room temperature in the mixed solvent. The fact that the molecular structure of the mixed solvent does not change significantly when it is cooled to −12 °C, indicating a dynamic dissolution mechanism.

## II. THEORY AND METHODS

### A. Neutron scattering method and small angle neutron diffractometer for amorphous and liquid samples (SANDALS)

The small angle neutron diffractometer for amorphous and liquid samples (SANDALS) is a diffractometer at the ISIS Neutron and Muon Source, a spallation neutron source. The scattering vector (Q) range of SANDALS is 0.1 Å^−1^ to 50 Å^−1^, which means that the space measurement range for the radial distribution function (RDF) of disordered materials is from sub-Angstrom distance resolution (∼0.1 Å) out to a maximum length scale of ∼30 Å. Facing a liquid methane moderator, SANDALS receives neutrons with wavelengths in the range 0.05–4.95 Å, and it has a resolution of 2% ΔQ/Q across most of its operating Q-range. The typical measurement time for a hydrogen-containing sample is about 6 h and shorter for a deuterium or non-hydrogen containing samples.

#### 1. Experiment samples

**FIG. 1.**
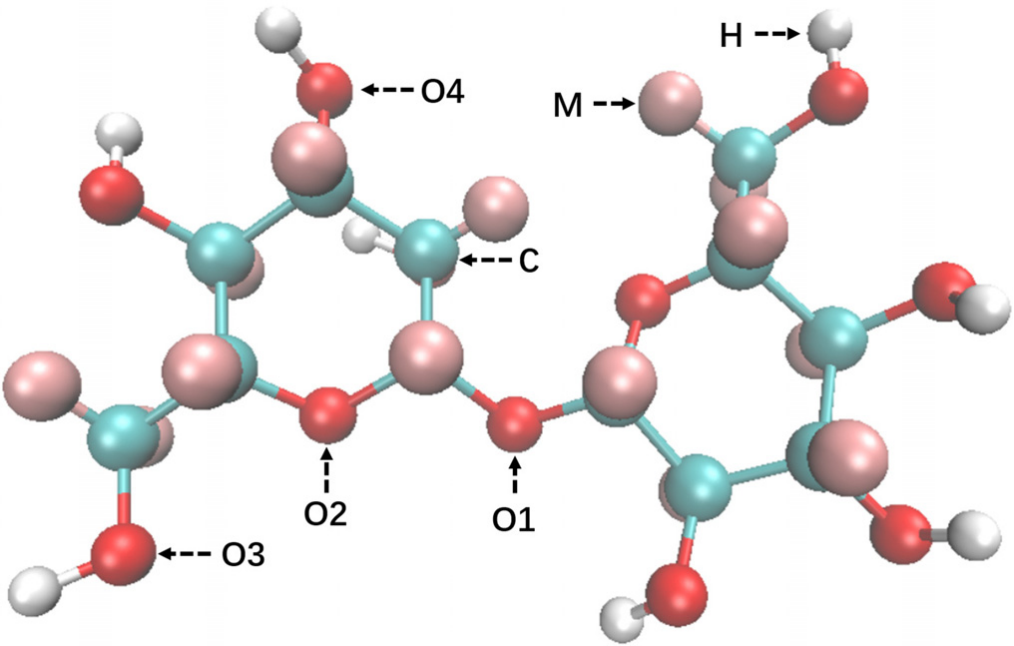
Structure of a trehalose molecule and its atomic labels used in EPSR simulation. All carbon atoms are labeled C; all hydrogen atoms connected to the oxygen atom are labeled H, and the remaining hydrogen atoms connected to the carbon atom are labeled M; the oxygen atom linking the two sugar rings is labeled O1, the oxygen atom on the sugar ring is labeled O2, the oxygen atom on the methyl group is labeled O3, and the oxygen atoms on the hydroxyl group connected to the sugar ring is labeled O4.

All of the hydrogenated trehalose, urea, and NaOH were obtained from Shanghai Aladdin Biochemical Technology Co. Ltd., and all of the deuterated water, NaOH, and urea were purchased from Sigma-Aldrich China. Their deuterium purities are higher than 99%. In this study, hydrogenated trehalose is used as a model molecule for cellulose. Its molecular structure is shown in Fig. 1, and its radius of gyration *R_g_* is about 4.0 Å. Trehalose is composed of glucose rings similar to that of cellulose, and its radius of gyration is in the detection range of SANDALS ($Q_{\text{min}}=0.1$Å^−1^).

Three kinds of samples, i.e., NaOH/urea/trehalose, NaOH/urea, and NaOH/trehalose aqueous solutions, with three different deuterium ratios were prepared. The labels, chemical components, and deuterium ratios are listed in Table I. The molar ratios of NaOH, urea, and H_2_O are all kept the same to those in Professor Lina Zhang's research.^5,8^ Each sample is measured at both 25 °C and −12 °C (or −10 °C, if the deuterated sample freezes at −12 °C), as well as the empty Ti/Zr sample cells used. Then, the data processing program package GudrunN for reducing total scattering data is used to subtract backgrounds, multiple scattering, and fix attenuations.

**TABLE I.** Sample labels, chemical components, deuterium ratios, and molar ratios of the samples.

Sample labels	NaUrH_2_O/HDO/D_2_O	NaTrH_2_O/HDO/D_2_O	NaUrTrH_2_O/HDO/D_2_O
Chemical component	NaOH urea water	NaOH trehalose water	NaOH urea trehalose water
Deuterium ratio	0.0/0.5/1.0	0.0/0.5/1.0	0.0/0.5/1.0
Molar ratio	NaOH:urea:trehalose:water ≈ 0.147 0:0.167 8:0.042 3:3.776 8		

### B. EPSR simulations

Empirical potential structure refinement (EPSR) is used to reconstruct the three-dimensional atomic structure based on neutron total scattering.^14–17^ EPSR is essentially a Monte Carlo simulation, where the force field used in EPSR simulations is divided into reference potential and empirical potential. The reference potential is used to maintain the known constraints, such as the molecular structure, atomic number density, and expected Lennard–Jones parameters for the individual molecular groups, while the empirical potential is used to drive the simulation toward the experimental data. EPSR will run continuously until the increase in empirical potential has no effect on the quality of the fits of the simulated structure factor to the experimental one.

The atom labels used for trehalose in EPSR are shown in Fig. 1. The O/H atoms of H_2_O, Na^+^, O/H atoms of OH^−^, and O/C/N/H atoms of urea are labeled as OW, HW, Na, ONa, HNa, OU, CU, NU, and HU, respectively. The molecular structure of trehalose is established by the Jmol program first and then refined by the MOPAC-7 molecular orbital program with the Hamiltonian method AM1. Its molecule modeling, the L–J reference potential parameters, and charges are OPLS-AA^18–23^ and listed in Table II. (Data accuracy is retained to three decimal places.) The cubic EPSR simulation box size, atomic number density, and numbers of molecules contained in each sample are listed in Table III. (To show the difference, the atomic density is accurate to six digits after the decimal point.)

**TABLE II.** Lennard–Jones (L–J) reference potential parameters and charge (q) of atoms used in EPSR.

Atom label	OW	HW	Na	ONa	HNa	CU	OU	NU
*ε* (KJ/mol)	0.650	0.000	0.125	0.251	0.184	0.439	0.878	0.711
*σ* (Å)	3.166	0.000	2.500	2.750	1.443	0.375	2.960	3.250
q (e)	−0.848	0.424	0.679	−1.103	0.424	0.142	−0.390	−0.542
Atom label	HU	C	O	O1	O2	O3	H	M
*ε* (KJ/mol)	0.000	0.276	0.711	0.586	0.586	0.711	0.050	0.121
*σ* (Å)	0.000	3.500	3.100	3.100	2.900	3.100	1.700	1.700
q (e)	0.333	0.258	−0.500	−0.500	−0.500	−0.500	0.301	0.000

**TABLE III.** Cubic simulation box size, atomic number density, and number of molecules used in EPSR.

Sample labels	NaOH	Urea	Trehalose	Water	Box size (Å)	Density (atoms/Å^3^)
NaUrH_2_O	147	168	…	3777	50.162 0	0.103 915
NaTrH_2_O	147	…	42	3777	50.499 8	0.106 083
NaUrTrH_2_O	147	168	42	3777	52.104 7	0.106 080

## III. RESULTS

**FIG. 2.**
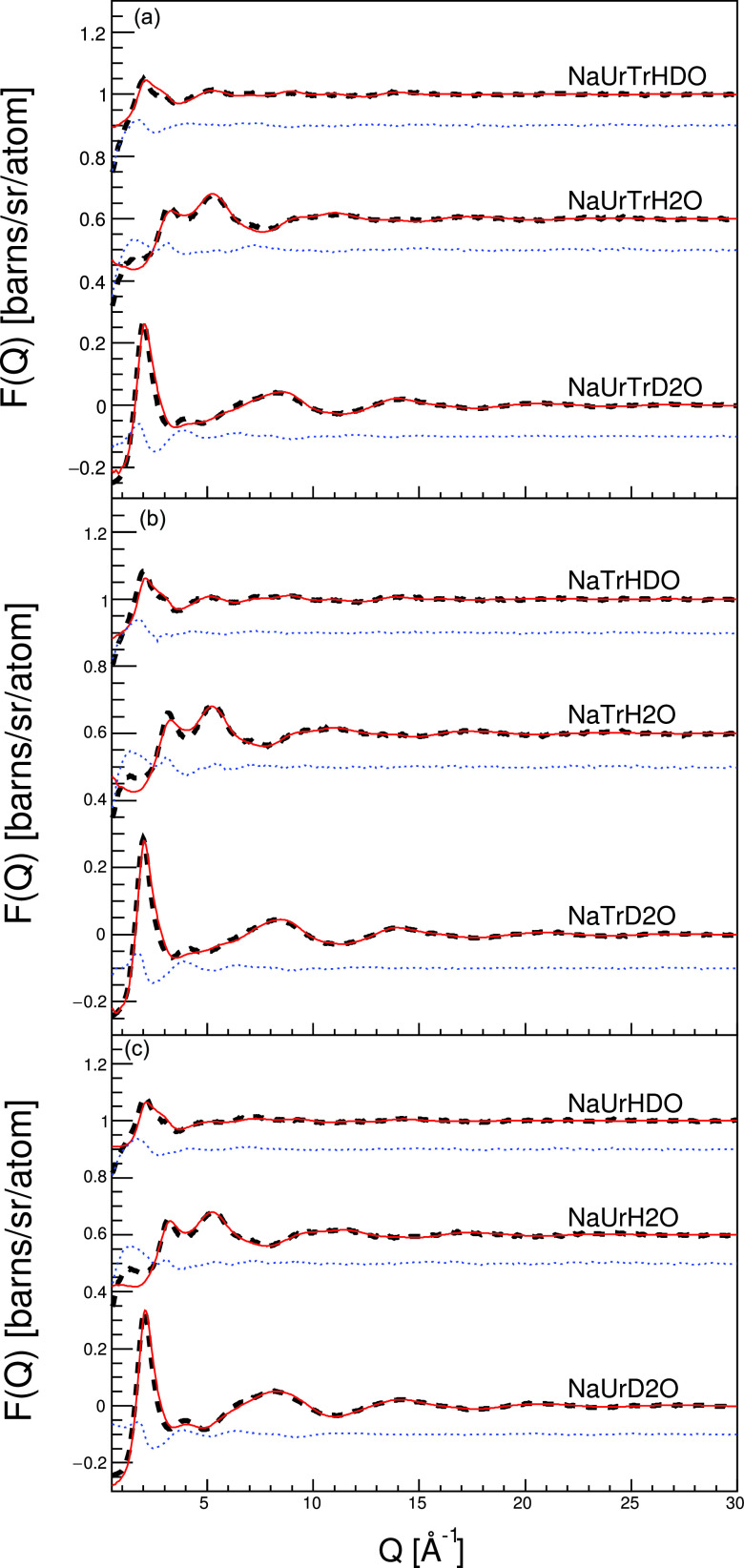
Experimental neutron total scattering profiles (dashed black lines), the EPSR fitted neutron structure factors (red lines), and their differences (blue dots). (a) Trehalose in NaOH/urea aqueous solution, (b) trehalose in NaOH aqueous solution, and (c) urea/NaOH in water. Data have been offset for clarity.

The real space most probable all atom positions are obtained from the EPSR simulation refined against the neutron scattering data. The simulated scattering curves for each of the different isotopic substitutions can be calculated from the EPSR simulation box by a sum of all the atom–atom pair correlations, weighted by their scattering lengths and concentrations,

$$\begin{array}{c} F(Q)=\sum_{\alpha =1}^{N}c_{\alpha }b_{\alpha }^{2}+\sum_{\alpha =1,\beta \geq \alpha }\left(2-\delta_{\alpha \beta }\right)c_{\alpha }c_{\beta }b_{\alpha }b_{\beta }\\ \times \{4\pi \rho \int_{0}^{\infty }r^{2}(g_{\alpha \beta }(r)-1)\frac{\;\text{sin}\;(Qr)}{Qr}dr\}, \end{array}$$ (1)

where Q=4π/λ sin(θ/2) is the scattering vector, *λ* is the neutron wavelength, *θ* is the scattering angle, $\sum_{\alpha }c_{\alpha }b_{\alpha }^{2}$ is the self-scattering background, $(2-\delta_{\alpha \beta })c_{\alpha }c_{\beta }b_{\alpha }b_{\beta }$ are the weighted factors of different partial structural factors, $c_{\alpha /\beta }$ and $b_{\alpha /\beta }$ are the atom ratio and scattering length of atoms with types α/β, respectively, and $g_{\alpha \beta }(r)$ is the RDF between atom types *α* and *β*. Figure 2 shows the scattering curves from the neutron experiment and the refined simulation, showing a good level of fit. We can, therefore, have confidence that the produced three-dimensional ensemble from the EPSR simulation represents the most probable structure of the system that is consistent with the available data.

The hydration layer around trehalose is critical for it to dissolve in the complex fluid.^22,23^ Specific attention should be paid to the area around the four kinds of electronegative oxygens of trehalose (O1, O2, O3, and O4 in Fig. 1, and called Os, when referring to the whole set of oxygens—regardless of their position within the molecule.), which provide the hydrophilic targets. O1 links the two glucopyranose rings, and O2 forms part of the glucose rings, so they are located inside the trehalose molecule, while O3 and O4 belong to hydroxyl groups that are located on the periphery. In this study, the most probable all-atom positions of solvent molecules with these four kinds of oxygens have been investigated through their radial distribution functions, ${\text{g(r)}}_{\alpha \beta }$,

$$g{\left(r\right)}_{\alpha \beta }=\frac{\Delta n_{\alpha \beta }(r)}{4\pi r^{2}\Delta r\rho_{\alpha }},$$ (2)

where $\Delta n_{\alpha \beta }(r)$ represents the atom number of atomic species *α* in a spherical shell with radius r and thickness Δr around an atom of atomic species *β*, $4\pi r^{2}\Delta r$ is the volume of the spherical shell, and $\rho_{\alpha }$ represents the atomic density of atomic species *α* in the system. The ${\text{g(r)}}_{\alpha \beta }$ reflects the relationship between the local density of the atomic number and the overall average density. If it is greater than 1, it means that the atom density here is denser than the average; if it is less than 1, the atomic density here is lower than the average.

**FIG. 3.**
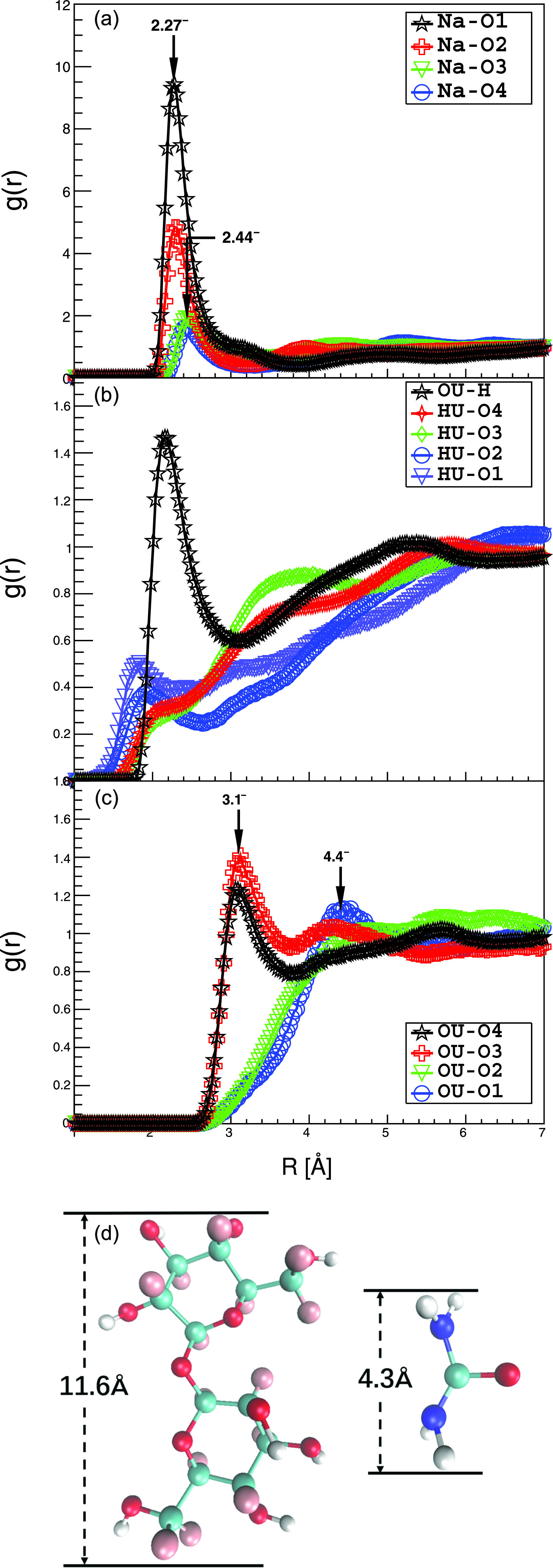
RDF distributions between the solvent atoms and the solute hydrophilic atoms. (a) g(r)s between Na^+^ and Os of trehalose; (b) g(r)s between urea and Os or H of trehalose; (c) g(r)s between oxygen of urea and Os of trehalose; (d) schematic diagram of the approximate molecular size of trehalose and urea. The arrows are used to guide the eye.

We focus on the effect of NaOH in the dissolution process first. In previous NMR studies, it was found that NaOH hydrate forms a new hydrogen bond system with cellulose. The role of the hydrate of Na^+^ is to stabilize the hydrophilic hydroxyl groups in cellulose.^4,5,24^ Here, we can visualize the atomic position of Na^+^. The position of the first peak in ${\text{g(r)}}_{\alpha \beta }$, g_1_(r) means the nearest most probable distance between the two kinds of atoms, and its magnitude represents the ratio of the local atomic number density to the average density at this most probable position. All together, there are 36 different ${\text{g(r)}}_{\alpha \beta }$ of solvent atoms with the Os of trehalose. Only the four $g_{1}(r){}_{\mathrm{Na-Os}}$ are larger than 1 [Fig. 3(a)]. The g_1_(r)s of the Na^+^ around O1 and O2 of trehalose have very sharp peaks at about 2.27 Å, so Na^+^ is accumulated around O1 and O2. O1 and O2 are at the joint of two glucopyranose rings, so the space around them is relatively confined. Only small molecules or ions, such as H_2_O and Na^+^, can occupy these spaces. On the other hand, O3 and O4 are located on the periphery of trehalose, so any molecule could access them. Their g_1_(r) peaks with Na^+^ are located at 2.44 Å, which are further away from the 2.27 Å peaks. This result shows that Na^+^ can be very close to the glucose ring of cellulose, thereby breaking its intra- and inter-molecular hydrogen bonds.

Now we look into the effects of urea on the dissolution process. Urea may be either proton donor or acceptor to form hydrogen bonding, so we compare all of its g(r)s with trehalose, i.e., g(r)${}_{\text{HU}-\text{Os}}$ as proton donor and g(r)${}_{\text{OU}-H}$ as proton accepter [Fig. 3(b)]. A urea molecule is composed of 8 atoms through covalent bonds, so it is much larger than a Na^+^ ion [Fig. 3(d)]. This prevents urea molecules from penetrating into the trehalose structure, as required to act as proton donor, resulting in the partial g(r) being <1. Since urea may also play the role of proton acceptor to form hydrogen bonding. We also study its g(r) with H of trehalose. Figure 3(b) also demonstrates that g_1_(r) between OU and H (trehalose) is larger than 1.0. Hydrogen bonding is defined in terms of a short-range donor–acceptor distance (3.0 Å) and an almost linear bond angle (within ±20°). No such hydrogen bonding interactions are found.

To quantify the distribution of urea molecules around trehalose, we look at the g(r) between OU and the four Os of trehalose [Fig. 3(c)]. It shows that urea can only locate around the periphery of trehalose. g_1_(r)s of OU-O4 and OU-O3 are larger than 1, i.e., 1.2 and 1.4, respectively. They are located around $r_{1}=3.1$ Å, which are larger than the g_1_(r) peak positions of Na^+^-O4 and Na^+^-O3 ($r_{1}=2.44$ Å). On the other hand, g_1_(r)s of OU-O1 and OU-O2 are closer to 1.0 at 4.4 Å, which are also further away from the g_1_(r)s of Na^+^-O1 and Na^+^-O2 at 2.27 Å. All of these show that urea cannot interact with trehalose directly.

**FIG. 4.**
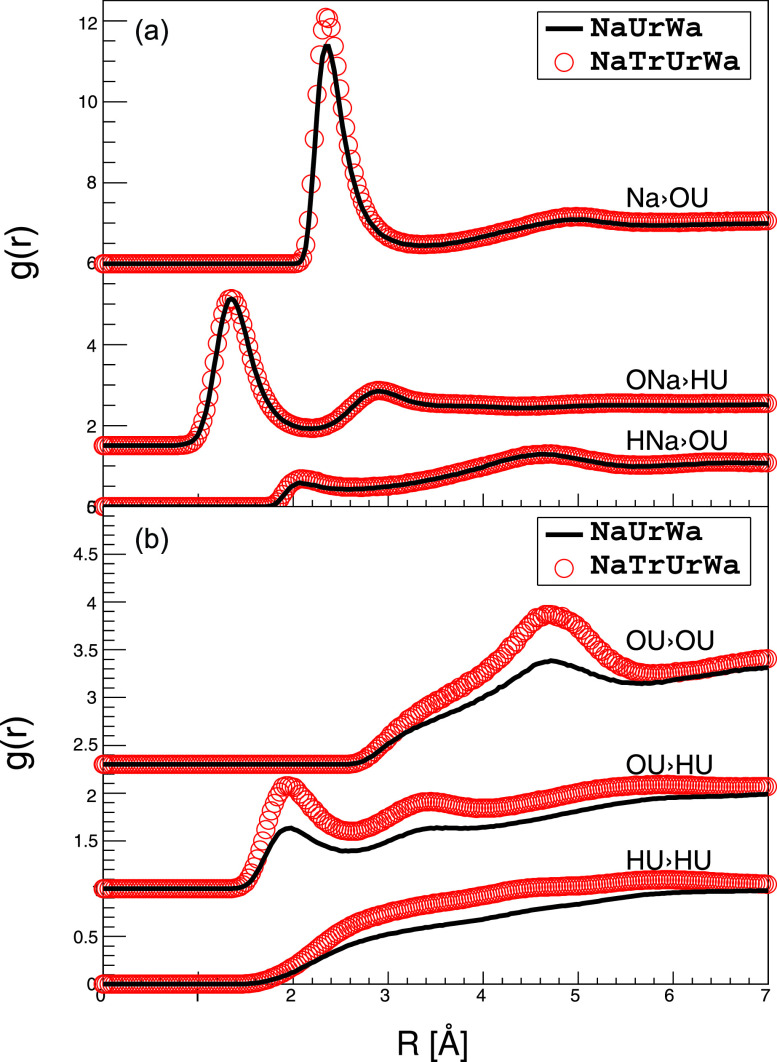
RDF distributions of NaOH/urea with (red circles) and without (black lines) trehalose. (a) ${g(r)}_{\text{Na}-\text{OU}}$, ${g(r)}_{\text{ONa}-\text{HU}}$, and ${g(r)}_{\text{HNa}-\text{OU}}$; (b) ${g(r)}_{\text{OU}-\text{OU}}$, ${g(r)}_{\text{OU}-\text{HU}}$, and ${g(r)}_{\text{HU}-\text{HU}}$.

Comparison of the g(r)s of NaOH/urea aqueous solution with and without trehalose further strengthens this picture. There are no significant differences on g(r)s between NaOH and urea [Fig. 4(a)], while the peak heights of g_1_(r) between urea and urea increase by a factor of 1.4 [Fig. 4(b)] in the presence of trehalose. Here, the volume fraction of urea decreases by a factor of about 12%, and the atom number of urea inside its first g_1_(r) shell (r < 5.8 Å) increases by about 16% after trehalose is added. As discussed in the previous experimental results, urea cannot enter the interior of trehalose molecule, but small molecules, such as water, Na^+^, and OH^−^, do penetrate. On the one hand, trehalose excludes a volume that urea cannot access; on the other hand, NaOH is not displaced by the presence of trehalose in the same way. Therefore, g_1_(r)${}_{\text{urea}-\text{urea}}$ increases a factor of 1.4 with the presence of trehalose, and g_1_(r)${}_{\text{urea}-\text{NaOH}}$ does not change. Nevertheless, the position of g_1_(r)${}_{\text{OU}-\text{OU}}$ is confined by the inter-hydrogen bond length of urea.

**FIG. 5.**
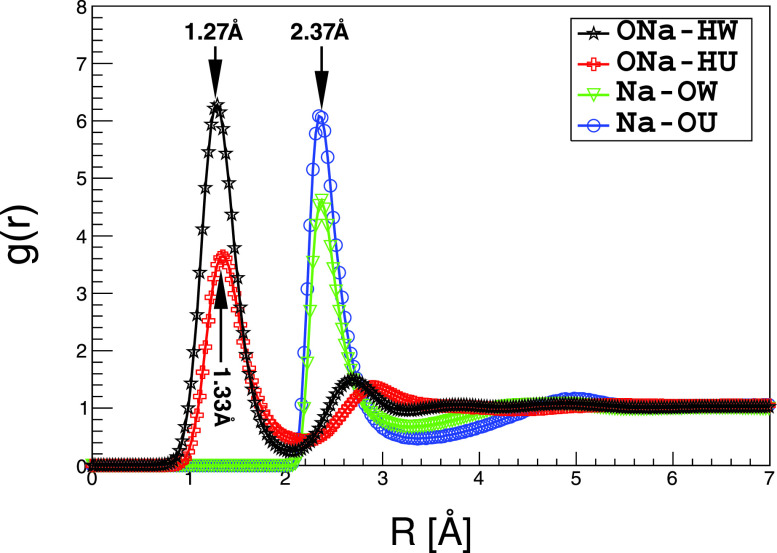
RDF comparison between NaOH-urea and NaOH-H_2_O.

Urea cannot interact with trehalose directly. Therefore, understanding the interactions between urea and the Na^+^-dominated hydration shell of trehalose is critical. Jiang *et al.* have used NMR and cryo-TEM to study the dissolution mechanism of cellulose in NaOH/urea aqueous solutions. Their NMR observation indicates that OH^−^ of NaOH directly interacts with the amino groups of urea through hydrogen bonds, and their cryo-TEM experiment confirms the existence of a sheath like inclusion complex around cellulose, which may be composed of urea hydrates. They concluded that it is the hydrogen bonding between OH^−^ of NaOH and urea that connects urea to the hydration shell.^4,12^ Here, we compare the interaction of NaOH with water and urea (Fig. 5). g_1_(r)${}_{\text{HW}-\text{ONa}}$ at 1.27 Å is 1.8 times larger than ${g(r)}_{\text{ONa}-\text{HU}}$ at 1.33 Å, confirming that NaOH hydrates water stronger than urea. On the contrary, g_1_(r)${}_{\text{Na}-\text{OU}}$ is 1.4 times larger than g_1_(r)${}_{\text{Na}-\text{OW}}$ at 2.37 Å, showing that Na^+^ interacts with urea more strongly than with water. From this, we conclude that urea interacts with trehalose via Na^+^ bridging.

**FIG. 6.**
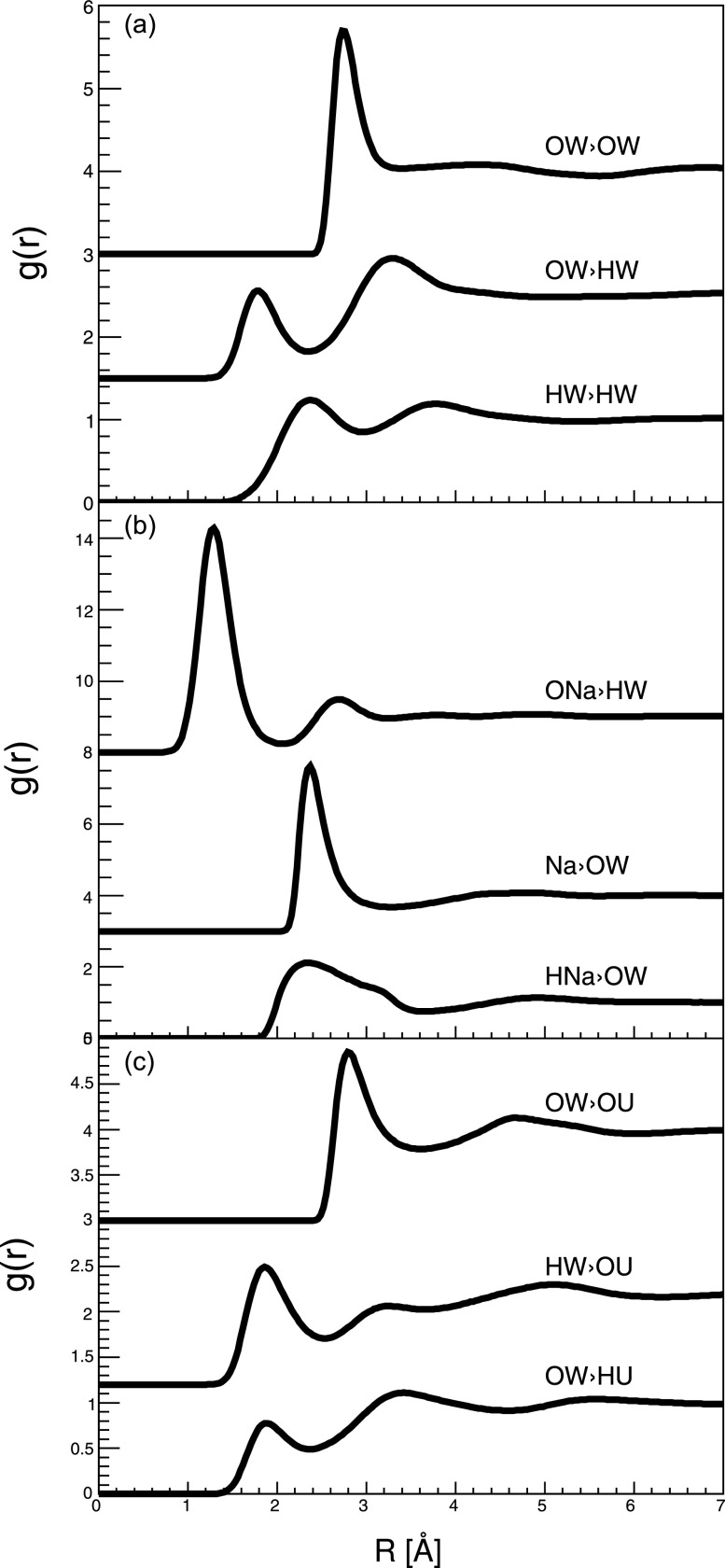
RDF distributions between some solvent atoms. (a) Water–water correlations: ${g(r)}_{\text{OW}-\text{OW}}$, ${g(r)}_{\text{OW}-\text{HW}}$, and ${g(r)}_{\text{HW}-\text{HW}}$. (b) Water–NaOH correlations: ${g(r)}_{\text{ONa}-\text{HW}}$, ${g(r)}_{\text{Na}-\text{OW}}$, and ${g(r)}_{\text{HNa}-\text{OW}}$. (c) Water–urea correlations: ${g(r)}_{\text{OW}-\text{OU}}$, ${g(r)}_{\text{HW}-\text{OU}}$, and ${g(r)}_{\text{OW}-\text{HU}}$.

We finally investigate the effects of water, the main component of the complex fluid. Figure 6 shows the RDF distributions of water–water, water–NaOH, and water–urea in the complex fluid. A cosolvent can influence the solubility of a solute via direct or indirect interaction. In the former case, the cosolvent binds to the solute molecule directly;^25^ while in the latter case, it interacts with the solvent (water in this case) to form a new static or dynamic structure,^26^ which changes the solubility of the solute. Pure liquid water has a short-range ordered tetrahedral structure, without any long-range order.^27–29^ In the presence of NaOH and urea, this short-range ordered is disturbed. Compared with the results in literature about water structure, the second peak in g_OW-OW_(r) is depressed in Fig. 6(a).^30^ This is similar to the results in the literature.^18–21^ In the complex fluid, the cosolvent (NaOH and urea) distributions around water molecules [Figs. 6(b) and 6(c)] are similar to literature results on pure NaOH and urea aqueous solution in literatures;^18–21^ for example, the first peak on g_Na-OW_(r) is at about 2.4 Å; the first and second peaks on g_HW-OU_(r) are at 1.9 and 3.1 Å, respectively; and so on. Therefore, both direct and indirect interactions exist in the complex fluid. Na^+^ directly interacts with trehalose, and the structure of water is disturbed in the presence of NaOH and urea.

**FIG. 7.**
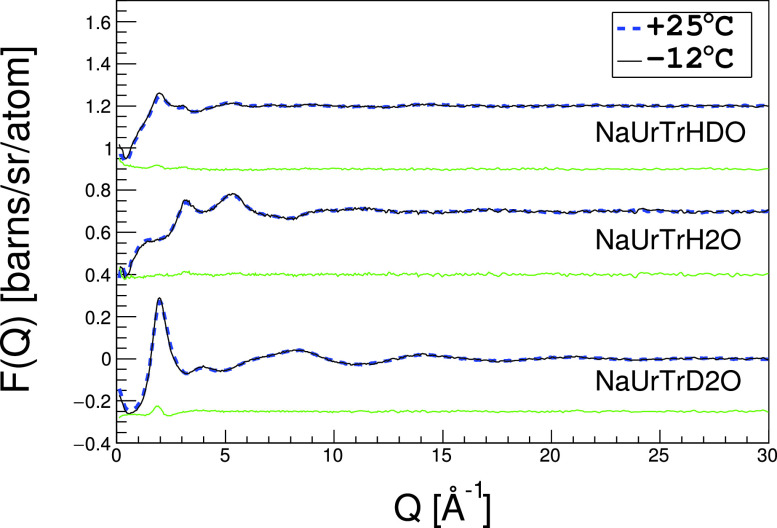
Neutron total scattering profiles in the complex fluid at 25 °C, −12 °C, and the difference between them (green lines).

Cellulose dissolves in the mixed solvent at around −12 °C but precipitates at room temperature. We, therefore, investigated the temperature dependence of structural variations in the complex fluid. Figure 7 shows the neutron scattering profiles in the complex fluid at +25 °C and −12 °C. We find that temperature-dependent changes to the atomistic structures are negligible. At −12 °C, the hydration shell around trehalose has almost the same static atomic structure but is dynamically more stable than that at room temperature. It thereby strongly prevents the re-aggregation of already dissolved cellulose.^12^

**FIG. 8.**
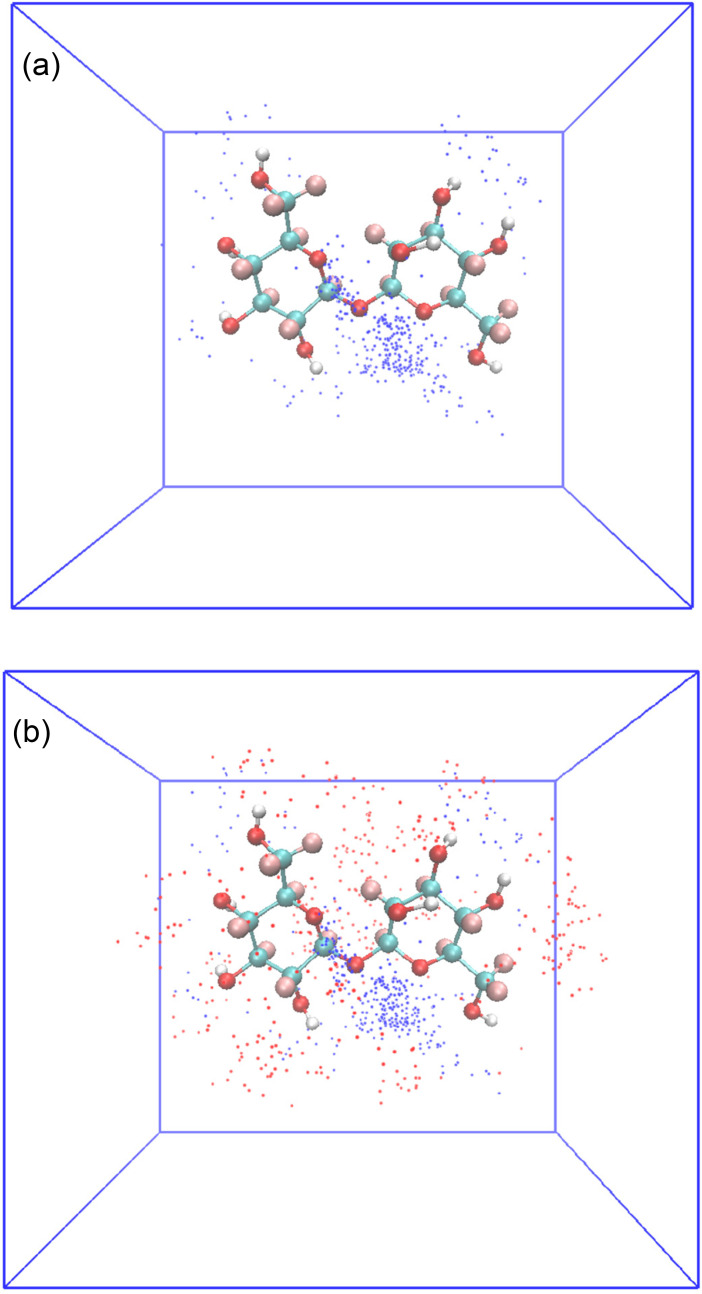
The schematic diagram of the accumulation of sodium ions and urea molecules around trehalose. (a) Na^+^ ion distribution (blue dots) around a trehalose molecule. (b) Distribution of Na^+^ and the oxygen atoms of urea (red dots) around a trehalose molecule.

The distribution probability of Na^+^ and urea in the hydration shell of trehalose derived from EPSR is depicted in Fig. 8. The molar ratio of Na^+^:OU:trehalose is 147:168:42, so the resultant schematic diagram is the accumulation of 100 frames to get good statistics. As shown in the picture, small blue Na^+^ ions directly interact with the Os of trehalose. Most of them are accumulated around O1 and O2. Then, large red urea molecules bind to its surface to form the dynamic hydration shell.

## IV. CONCLUSIONS

Trehalose has been used as a model molecule to investigate the mechanism of rapid dissolution of cellulose in an NaOH/urea aqueous solution at two different temperatures. With the combination of neutron total scattering and EPSR simulation, the dissolution mechanism is understood at an atomic scale for the first time. We find that NaOH, urea, and water work cooperatively to dissolve trehalose. NaOH directly interacts with trehalose, and Na^+^ accumulates around the electronegative oxygens of the glucose rings. Urea then binds to trehalose via Na^+^ bridging. We further investigated the effects of temperature on the dissolution process. Structural differences between the system at −12 and 25 °C are negligible. We, therefore, conclude that previous observations of an increased cellulose solubility at low temperature are caused by changes to the dynamics in the fluid. Reduced mobility at low temperatures stabilizes the hydration shell around the cellulose molecules, preventing cellulose from re-aggregating, thereby aiding the dissolution process.

We predict that smaller alkali, such as LiOH, may dissolve trehalose even better. Therefore, neutron scattering experiments have been expanded to studying the atomic structure of trehalose, LiOH/urea, and KOH/urea aqueous solutions. Results from these experiments will be presented in a follow-up study.

## Data Availability

The data that support the findings of this study are openly available in ISIS Neutron and Muon Source Data at http://doi.org/10.5286/ISIS.E.RB1820090, Ref. 31.
